# Higher processing repeatability of myocardial flow reserve calculated using net retention model compared to one compartment model in SPECT studies

**DOI:** 10.1038/s41598-024-67474-z

**Published:** 2024-07-19

**Authors:** Pawel Cichocki, Anna Plachcinska, Michal Blaszczyk, Zbigniew Adamczewski

**Affiliations:** 1 Department of Nuclear Medicine, Medical University of Lodz, Lodz, Poland; 2 Department of Quality Control and Radiological Protection, Medical University of Lodz, Lodz, Poland

**Keywords:** CZT SPECT, Myocardial blood flow, Myocardial flow reserve, Repeatability, Ischaemia, Radionuclide imaging

## Abstract

Dynamic assessment of myocardial blood flow (MBF) and myocardial flow reserve (MFR) provides additional information that can improve diagnostic accuracy of radionuclide myocardial perfusion imaging in some clinical situations. This study assessed processing repeatability of these parameters calculated using two models—net retention (RET) and one compartment (1CM) in dynamic SPECT studies, using the latest version of Corridor 4DM software (v2024). Data of 107 patients were analyzed retrospectively (57 of whom were assessed in our previous study using 4DM v2015). Dynamic SPECT studies were carried out using a routine two-day rest-dipyridamole protocol. Data was processed in 4DM v2024 twice by one operator and once by another operator. Automatic heart image positioning during post-processing in 4DM v2024 was significantly improved compared to v2015, reducing the number of studies requiring extensive manual corrections from 41 to 12%. This significantly improved interobserver processing repeatability of MFR values in RCA territory compared to our previous study using v2015—from *r* = 0.67 to 0.85 (*p* = 0.0034). Interobserver processing repeatability of MBF and MFR in all 107 patients was significantly better in RET model compared to 1CM model. In conclusion, RET model is more reliable for calculating MBF and MFR values based on dynamic SPECT studies.

## Introduction

Myocardial perfusion imaging (MPI) is one of the most commonly used procedures in nuclear medicine. Its usefulness in the non-invasive diagnosis of coronary artery disease (CAD) is well documented. However, it has some limitations—including lower sensitivity in detecting multivessel CAD. In these cases, dynamic assessment of myocardial blood flow (MBF) and myocardial flow reserve (MFR) can be helpful. PET studies with ^82^Rb, ^13^N-ammonia or ^15^O-water are considered the gold standard in radionuclide assessment of MBF and MFR, however their availability is limited^1^. This makes it difficult to use MBF and MFR in PET in routine diagnostic procedures and in practice it is often limited to research studies conducted in well-equipped clinical hospitals. The latest advances in nuclear medicine, in the form of semiconductor detectors (cadmium-zinc-telluride—CZT) and dedicated cardiac gamma cameras as well as ring detector systems, allow dynamic assessment of MBF and MRF in SPECT studies using commonly available, 99 m-technetium-labelled radiopharmaceuticals such as ^99m^Tc-MIBI and ^99m^Tc-tetrofosmin. This significantly increases the availability and reduces the cost of determining these parameters, offering the potential to include them in routine cardiac diagnosis. However, SPECT gamma cameras and technetium-labelled radiopharmaceuticals have certain limitations.

In our previous work evaluating processing repeatability of MBF and MFR in dynamic studies performed using CZT Discovery NM530c cardiac gamma camera (GE Healthcare, Chicago, IL, USA), we found relatively poor interobserver repeatability of MFR values in RCA vascular territory^2^. So far, such a problem was not reported on the D-SPECT camera (Spectrum Dynamics, Israel) or in preliminary reports regarding the determination of MBF and MFR using the latest models of SPECT cameras with full-ring CZT detector configuration—Veriton (Spectrum Dynamics, Israel) and Starguide (GE Healthcare, Chicago, IL, USA)^3^. This issue was not reported in PET studies either, where repeatability of MBF and MFR values is excellent, due to significant degree of automation of data processing^4^. We attributed worse processing repeatability in our studies to poor automatic positioning of heart images in the Corridor 4DM software (INVIA, Ann Arbor, MI, USA), requiring frequent manual corrections. Other recent studies using Discovery NM530c camera also reached similar conclusions^5,6^, with Cuddy-Walsh et. al. also pointing out differences between the net retention (RET) and one compartment (1CM) models.

Each model calculates radiopharmaceutical retention rate based on time-activity curves (TAC), representing myocardial and blood pool radiopharmaceutical concentration, that are measured in dynamic PET or SPECT study. RET model is based on a simpler formula, where correction of spillover from blood pool into myocardium is the same in each segment of the myocardium. 1CM model, more commonly used in PET studies, uses more complex calculations, including parameters accounting for both partial volume and spillover effects, that are corrected for each segment of the myocardium separately^7,8^. Formulas are presented in Supplementary materials.

So far, there is no clear consensus on the choice of model for calculating MBF and MFR parameters in SPECT studies, although results presented by Cuddy-Walsh et. al.^6^ suggest, that RET model may be preferred.

Due to the observed technical problems with automatic heart image orientation in Corridor 4DM software, we have established cooperation with INVIA. Based on patient data from our previous publication, software was modified to improve the automatic alignment of heart images. The following modifications were made to the algorithm to improve the automated left ventricle (LV) contouring for dynamic MBF and MFR studies: the starting point of the search for the LV was modified to the center of the field of view based on the placement of the heart in Discovery NM530c gamma camera model, and the search radius for fitting the contours was reduced, to limit the potential influence of extra-cardiac activity. The latest version of the Corridor 4DM v2024 has been made available to us for testing purposes—courtesy of INVIA.

The aim of this study is to assess processing repeatability of MBF and MFR in dynamic SPECT MPI studies calculated using two models—RET and 1CM in 4DM v2024.

## Material and methods

### Patient population

The study included 119 patients with symptoms of CAD qualified for invasive diagnosis or with CAD diagnosed in a previously performed coronary angiography, who were referred for dynamic SPECT MPI with MBF and MFR assessment. 12 patients were excluded after quality control of the acquired images (5 due to patient motion artifacts, 3 due to incorrect bolus and 4 due to leak of radiopharmaceutical outside the vein). Since patient movement and significant respiratory motion can cause major artifacts during reconstruction of studies performed using Discovery NM530c camera, and since such motion occurs relatively rarely due to short image acquisition time, we opted to exclude all patients where such motion was detected during quality control instead of using software motion correction. Ultimately, 107 patients were included in the study, divided into 2 groups: group I consisted of 57 patients who had been evaluated in the previous publication using the older version of the Corridor 4DM software (v2015)^2^, while group II consisted of 50 new patients. Basic demographic and clinical data of included patients is summarized in Table 1.

**Table 1:** Basic demographic and clinical data of examined patients with comparison of Group I and Group II using Mann–Whitney U test and t-test.

	*N*	Male	Female	Age [median]	BMI [median]	Post infarction	PCI	CABG	Diabetes
All patients	107	68	39	45–84 [66]	17–40 [27]	47 (44%)	54	1	25
Group I	57	34	23	48–84 [64]	17–38 [27]	18 (32%)	23	0	11
Group II	50	34	16	45–83 [67.5]	21–40 [27]	29 (58%)	31	1	14
Gr. I vs Gr. II		*p* = 0.404		*p* = 0.100	*p* = 0.979	***p* = 0.006**	***p* = 0.019**	*p* = 0.860	*p* = 0.925

Significant values are in bold.

### Dynamic SPECT acquisition

All studies were performed using Discovery NM530c camera. All patients were examined according to the same two-day REST-STRESS (dipyridamole) protocol as in our previous work^2^. Patient preparation included discontinuing medications and avoiding foods and drinks that could potentially interfere with the study for an appropriate period. Patients were also advised to eat a meal with choleretic products (sandwich with hard-boiled egg or cheese) after radiopharmaceutical injection and before image acquisition, in order to accelerate clearance of the radiopharmaceutical by the liver.

On each day of the study, first 37 MBq of ^99m^Tc-MIBI were administered intravenously, for positioning on the gamma camera, with the heart in the center of the field of view of the detectors. After proper positioning, patients received 550 MBq of ^99m^Tc-MIBI in a manual rapid bolus injection, simultaneously with the start of image acquisition. Bolus injection was performed by the same person on each day of the study of each patient, to minimize potential variability. Dynamic list-mode image acquisition continued for 8 min. The stress part of the study additionally included a pharmacological test with intravenously administered dipyridamole (dose: 0.56 mg/kg body weight), performed after the patient was positioned on the camera. Bolus of radiopharmaceutical, with the same activity as in REST study, was administered 3 min after dipyridamole injection.

We did not use low-dose CT-based attenuation correction.

Acquired dynamic images were reconstructed using dedicated Xeleris Workstation software (GE Healthcare, Chicago, IL, USA). Dynamic list-mode images were reframed into 23 frames (15 × 6 s, 4 × 30 s, 4 × 60 s) and reconstructed according to the standard protocols provided by the camera vendor. At this stage, patient positioning on the camera and patient motion were evaluated.

**Figure 1:**
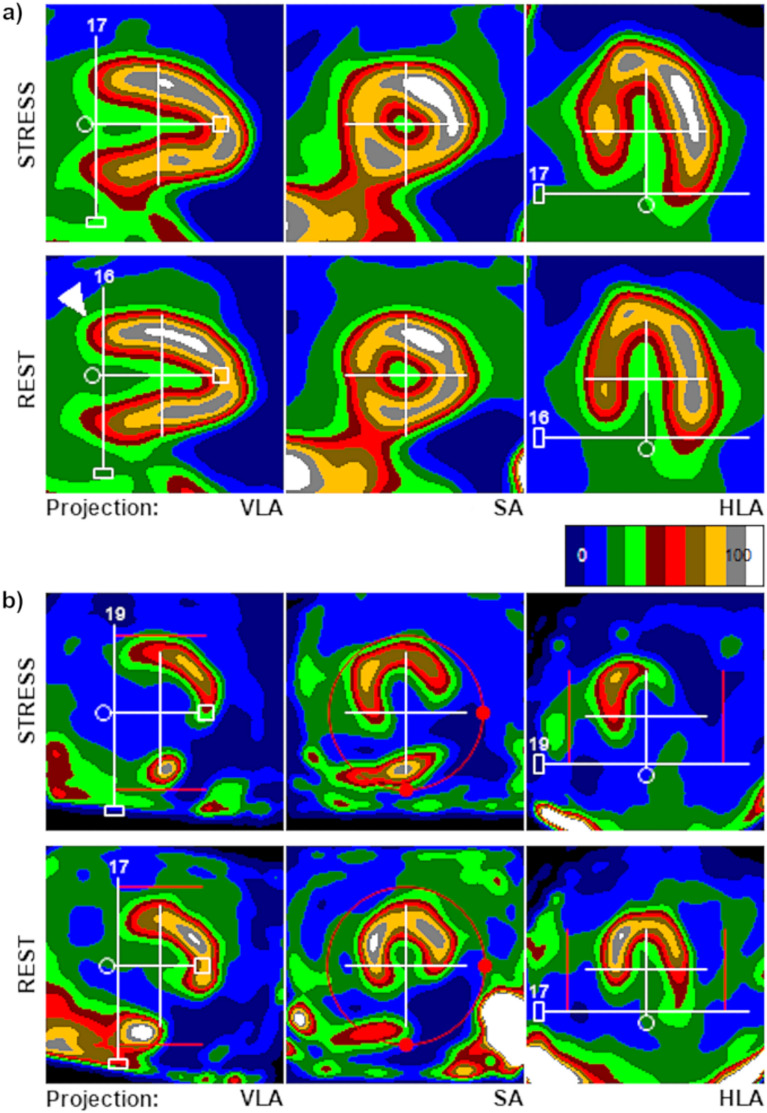
Examples of unmodified automatic image alignment—(**a**) correct position and orientation of image axis, with rest study needing only minor correction of the positioning of the last slice limit (valve plane, correct position marked with white arrow); (**b**) incorrect automatic image orientation that requires major manual adjustments. Note a complete lack of radiopharmaceutical uptake in inferior wall (post-infarction scar) combined with spots of relatively high blood pool activity in liver and intestines.

Reconstructed dynamic images were then imported to a stand-alone workstation running the test version of Corridor 4DM v2024, where they were processed by two independent operators with several years of experience in assessing dynamic SPECT MFR studies. Additionally, one of the operators processed all studies twice, 2 weeks apart. Study processing was performed using the same methodology as in our previous work^2^ and consisted of positioning the long axis of the heart horizontally, with first slice centered on the apex of LV, and last slice placed at the location of the mitral valve, crossing the basal part of the anterior wall of LV at 50% of its maximum activity (between light and dark red colors in a Step10 scale—Fig. 1a). Initial post-processing was performed automatically by the software. Then operator assessed the quality of automatic image alignment on a scale of 0–2 (where 0—correct setting, not requiring axis correction, 1—axis center set correctly, angle required minor corrections, 2—axis center set incorrectly, outside of the center of LV—Fig. 1b) and made manual corrections if necessary.

In the second stage of post-processing, operator carried out manual heart motion correction, adjusting individual image frames so that the activity in the heart ventricles overlapped the contour of the left ventricle as little as possible (focusing mainly on the first frames, where the bolus of activity flowing into the ventricles was best visible). The LV ROI was automatically placed at the intersection of the long axis of the left ventricle and the valve plane slice and was not manually modified.

Calculations of MBF and MFR values in each study were performed using RET and 1CM models, in the default configuration provided by the software developer. Switching between models took place after the entire post-processing was completed and did not involve any further modifications to the input data.

### Statistical analysis

Normality of the distributions was tested with Shapiro–Wilk test. Groups I and II were compared using Mann–Whitney U test (for independent, nonparametric variables) and independent samples t-test (for independent, parametric variables). Correlations of examined parameters, some of which were not distributed normally, were assessed using non-parametric Spearman’s rank correlation coefficient r. Bland–Altman plots were also used for selected, normally distributed parameters. F-test was used to assess the relationship between standard deviations used to draw Bland–Altman plots. The significance of differences between mean and standard deviation values of measured parameters was assessed using paired samples t-test and F-test, respectively. Correlation coefficients of these parameters were compared using paired samples z-test (for dependent variables) and independent samples z-test (for comparison of group I and II). Precision of selected measurements was expressed as standard deviation of percentage difference of assessed values, for comparison with other publications using such parameter^5,6,9^. In all analyses, statistical significance was considered to be achieved when *p* ≤ 0.05. Calculations were carried out using Statistica v13.3 (StatSoft Polska, Poland) and LibreOffice v7.5.3.2 (X86_64) (The Document Foundation, Berlin, Germany) software.

### Ethical approval

The study was conducted according to the guidelines of the Declaration of Helsinki. Due to retrospective character of the study, Scientific Research Ethics Committee at the Medical University of Lodz waived the requirement for approval (decision number: RNN/172/23/KE, 13.06.2023).

### Consent to participate

Informed consent was obtained from all individual participants included in the study.

## Results

The number of studies in which automatic image alignment required manual corrections in 4DM v2024 was much lower than in the previous version. Currently, in more than half of stress and rest studies (59%) no significant manual corrections were needed (the image was immediately set as shown on Fig. 1a). Previously, this situation occurred in only 25% of studies, and in 41% central point of the image axis was set completely outside LV and required significant manual corrections (as demonstrated in Fig. 1b.). In the 4DM v2024, this situation occurred only in 9% of studies in the same group of patients, and in 12% of studies of all 107 patients (Table 2).

**Table 2:** Quality of automatic image orientation and extent of necessary manual adjustments.

	4DM v2015		4DM v2024					
	Group I		Group I		Group II		ALL	
	Stress	Rest	Stress	Rest	Stress	Rest	Stress	Rest
Little to no adjustments needed	23 40%	5 9%	37 65%	32 56%	30 60%	28 56%	67 63%	60 56%
Axis angle needed small corrections	19 33%	20 35%	15 26%	20 35%	13 26%	13 26%	28 26%	33 31%
Axis center needed manual repositioning	15 26%	32 56%	5 9%	5 9%	7 14%	9 18%	12 11%	14 13%

Next, MFR values for the entire myocardium (TOT) and individual vascular territories (LAD, LCx and RCA) in group I obtained in our previous work using 4DM v2015, were compared with values from the same studies processed in the same way by the same operators in 4DM v2024. In 4DM v2024, mean values of MFR were generally lower than in v2014. In RCA territory this difference was significant (2.11 ± 0.71 vs 1.83 ± 0.63, *p* = 0.035) while in LAD territory and in whole myocardium the differences were close to statistical significance (2.08 ± 0.69 vs 1.88 ± 0.66, *p* = 0.082 for LAD and 2.12 ± 0.70 vs 1.83 ± 0.59, *p* = 0.058 for TOT). Difference in LCx territory was insignificant (2.16 ± 0.87 vs 1.93 ± 0.71, *p* = 0.108). Inter- and intraobserver repeatability metrics for MFR values obtained in two different versions of the software are summarized in Table 3. Correlation coefficient of MFR values obtained by two different operators in RCA territory in 4DM v2015 was significantly lower compared to values from two assessments by the same operator. This difference is no longer present in 4DM v2024, where there are no significant differences between correlations of parameters values from assessments performed by the same and different operators.

**Table 3:** Inter- and intra-observer (2 weeks apart) variability and Spearman’s rank correlation of values calculated using 1 compartment model in group I from study assessments performed in two different versions of 4DM (v2015 and v2024).

	4DM v2015		4DM v2024		Statistical significance of difference between versions		
	*r*	Difference (mean ± SD)	*r*	Difference (mean ± SD)	*r*	Mean	SD
	Different operators						
MFR LAD	0.91	− 0.10 ± 0.29	0.93	− 0.09 ± 0.23	*p* = 0.498	*p* = 0.820	*p* = 0.133
MFR LCx	0.90	− 0.10 ± 0.37	0.93	− 0.08 ± 0.27	*p* = 0.336	*p* = 0.740	***p* = 0.031**
MFR RCA	0.67	− 0.15 ± 0.55	0.85	− 0.09 ± 0.32	***p* = 0.023**	*p* = 0.532	***p* < 0.001**
MFR Total	0.89	− 0.13 ± 0.33	0.94	− 0.07 ± 0.20	*p* = 0.103	*p* = 0.152	***p* < 0.001**
	Same operator						
MFR LAD	0.88	− 0.12 ± 0.37	0.90	0.02 ± 0.25	*p* = 0.617	***p* = 0.019**	***p* = 0.004**
MFR LCx	0.91	− 0.12 ± 0.36	0.93	0.01 ± 0.26	*p* = 0.498	***p* = 0.035**	***p* = 0.017**
MFR RCA	0.84	− 0.13 ± 0.49	0.85	0.00 ± 0.31	*p* = 0.856	*p* = 0.074	***p* < 0.001**
MFR Total	0.91	− 0.10 ± 0.33	0.93	0.01 ± 0.23	*p* = 0.498	***p* = 0.031**	***p* = 0.005**

Significant values are in bold.

Group I and II were similar in terms of demographic and clinical data (with the exception of slightly larger number of patients with history of myocardial infarction and PCI in group II—Table 1). Also, correlations of MFR values calculated by different operators using 1CM and RET models in 4DM v2024 did not show statistically significant differences between group I and group II (Table 4).

**Table 4:** Detailed values of inter-observer processing variability (expressed as Spearman’s rank correlation r) of MFR values calculated using 1CM and RET models in group I and II in new version of Corridor 4DM software (v2024).

	1 compartment		Statistical significance of difference	Net retention		Statistical significance of difference
	Group I	Group II		Group I	Group II	
MFR LAD	0.926	0.874	*p* = 0.164	0.954	0.942	*p* = 0.552
MFR LCx	0.928	0.853	*p* = 0.062	0.976	0.955	*p* = 0.112
MFR RCA	0.846	0.795	*p* = 0.433	0.962	0.952	*p* = 0.551
MFR Total	0.938	0.899	*p* = 0.206	0.955	0.924	*p* = 0.179

**Figure 2:**
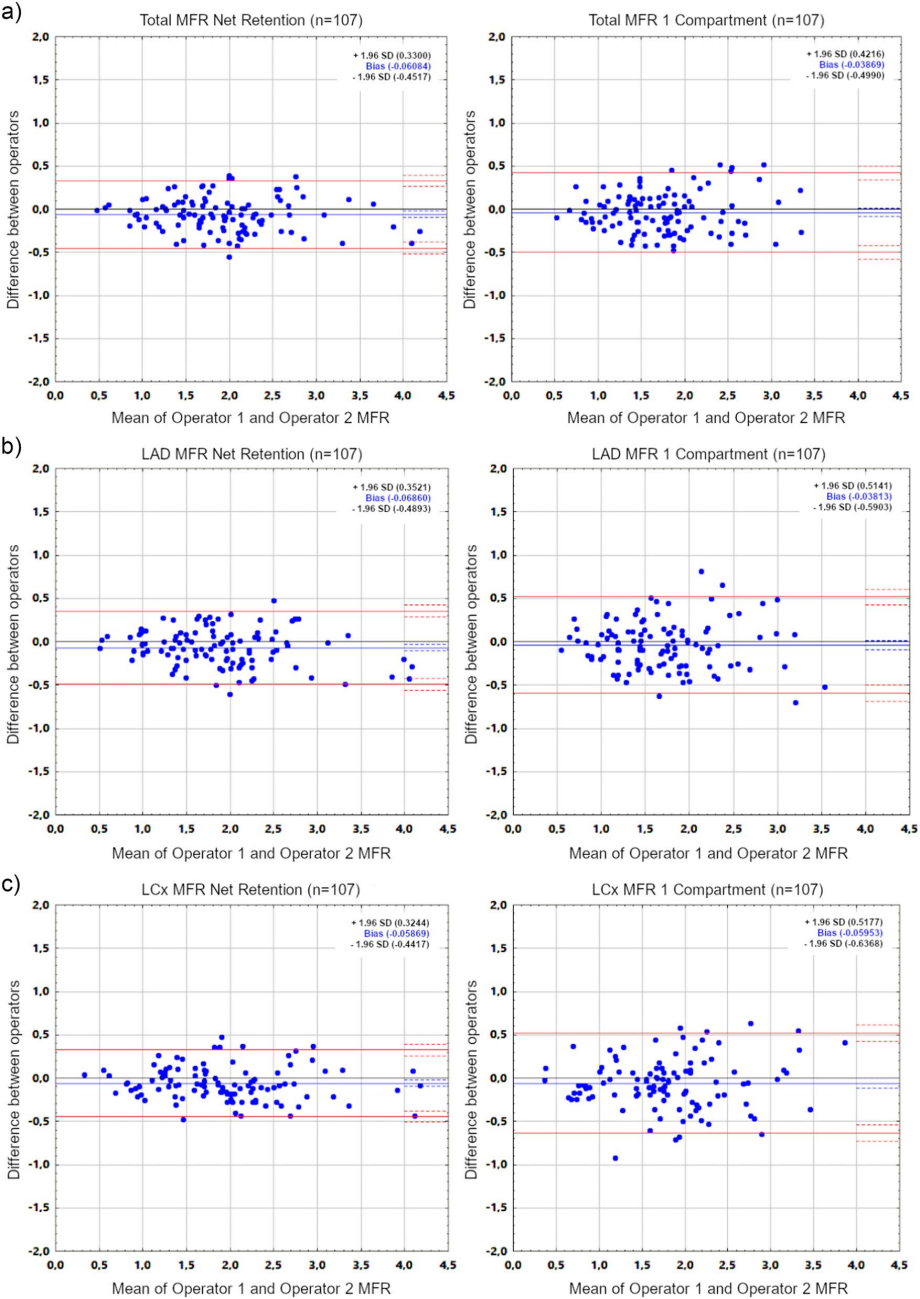

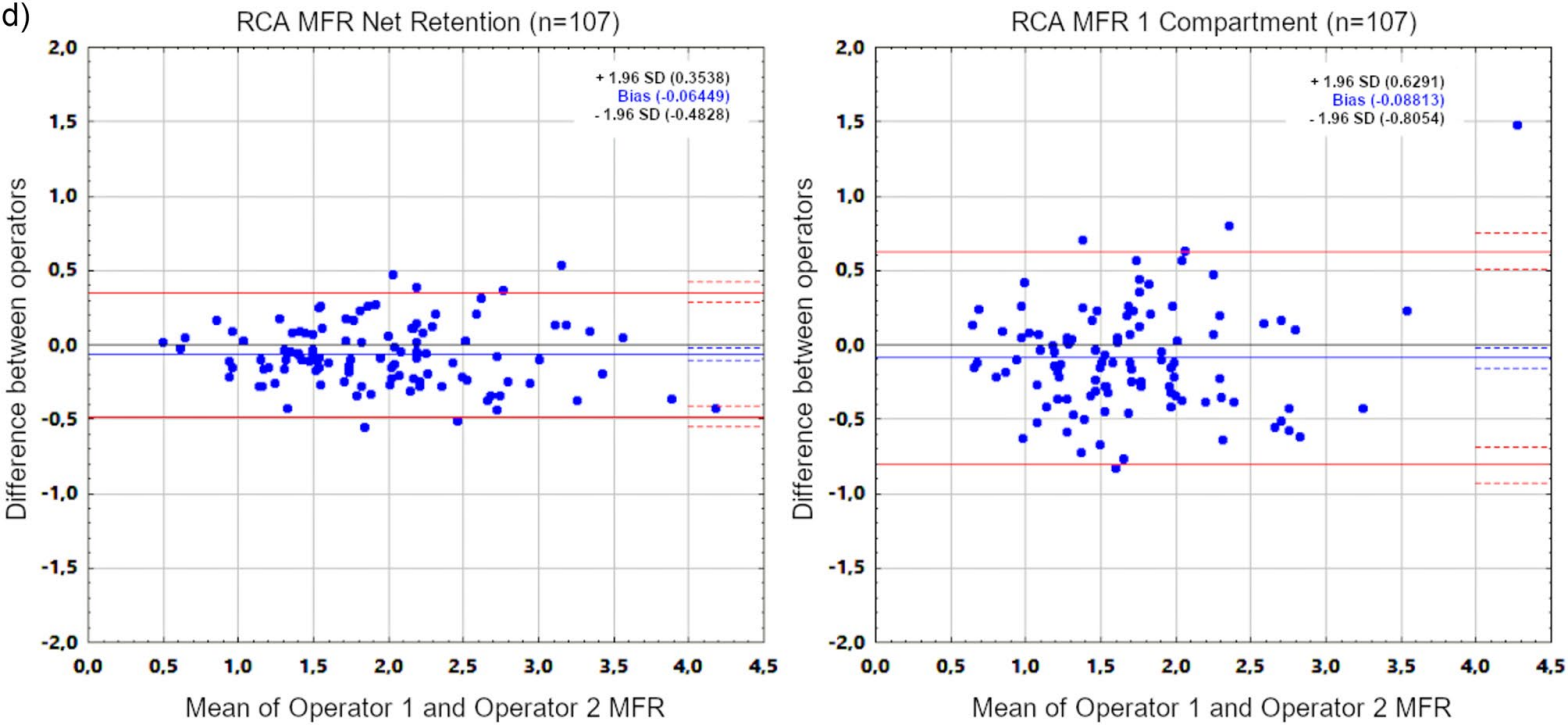
Bland–Altman plots for MFR values of all patients in whole myocardium (**a**) and all vascular territories (**b**–**d**) obtained by two operators using Net Retention and 1 Compartment models in 4DM v2024.

In the next step, studies of all patients processed by two operators using 1CM and RET models were assessed. Mean values obtained in 1CM and RET models by first operator and mean differences between them are presented in Table 5. Correlations and mean differences of assessed parameters obtained from study processing performed by two operators are summarized in Table 6. Additionally, Fig. 2 presents Bland–Altman plots of MFR values obtained in both models by two operators.

**Table 5:** Mean stress/rest MBF and MFR values calculated by first operator using 1 compartment and net retention models in all patients from study assessments performed in 4DM v2024, and mean differences between them.

		1 compartment	Net retention	Difference	Statistical significance of difference between means
		Mean ± SD	Mean ± SD	Mean ± SD	
STRESS MBF	LAD	1.91 ± 0.76	1.86 ± 0.73	0.05 ± 0.28	*p* = 0.604
	LCx	1.75 ± 0.67	1.79 ± 0.75	− 0.03 ± 0.26	*p* = 0.187
	RCA	2.08 ± 0.78	1.55 ± 0.63	0.52 ± 0.44	***p* < 0.001**
	Total	1.77 ± 0.69	1.71 ± 0.68	0.06 ± 0.22	***p* = 0.003**
REST MBF	LAD	1.17 ± 0.40	1.03 ± 0.36	0.14 ± 0.22	***p* < 0.001**
	LCx	1.01 ± 0.31	0.95 ± 0.32	0.06 ± 0.21	***p* = 0.004**
	RCA	1.28 ± 0.47	0.82 ± 0.30	0.45 ± 0.38	***p* < 0.001**
	Total	1.04 ± 0.31	0.92 ± 0.30	0.12 ± 0.15	***p* < 0.001**
MFR	LAD	1.69 ± 0.64	1.90 ± 0.76	− 0.21 ± 0.32	***p* < 0.001**
	LCx	1.80 ± 0.71	1.95 ± 0.80	− 0.14 ± 0.33	***p* < 0.001**
	RCA	1.71 ± 0.63	1.97 ± 0.75	− 0.25 ± 0.44	***p* < 0.001**
	Total	1.73 ± 0.60	1.93 ± 0.74	− 0.20 ± 0.29	***p* < 0.001**

Significant values are in bold. MBF values are in ml/min/g.

**Table 6:** Inter-observer processing variability and Spearman’s rank correlations of stress (s) and rest (r) MBF and MFR values calculated in all patients with 4DM v2024 using 1 compartment and net retention models.

	1 compartment		Net retention		Statistical significance of difference between models		
	Different operators		Different operators				
	*r*	Difference (mean ± SD)	*r*	Difference (mean ± SD)	*r*	Mean	SD
s MBF LAD	0.89	0.22 ± 0.34	0.96	0.08 ± 0.21	***p* < 0.001**	***p* < 0.001**	***p* < 0.001**
s MBF LCx	0.88	0.22 ± 0.32	0.94	0.11 ± 0.21	***p* = 0.010**	***p* < 0.001**	***p* < 0.001**
s MBF RCA	0.84	0.24 ± 0.45	0.95	0.08 ± 0.19	***p* < 0.001**	***p* < 0.001**	***p* < 0.001**
s MBF Total	0.91	0.19 ± 0.28	0.95	0.09 ± 0.20	***p* = 0.029**	***p* < 0.001**	***p* < 0.001**
r MBF LAD	0.84	0.18 ± 0.20	0.93	0.09 ± 0.12	***p* = 0.002**	***p* < 0.001**	***p* < 0.001**
r MBF LCx	0.81	0.19 ± 0.20	0.92	0.11 ± 0.12	***p* = 0.001**	***p* < 0.001**	***p* < 0.001**
r MBF RCA	0.72	0.23 ± 0.31	0.93	0.08 ± 0.11	***p* < 0.001**	***p* < 0.001**	***p* < 0.001**
r MBF total	0.84	0.16 ± 0.17	0.93	0.09 ± 0.11	***p* = 0.002**	***p* < 0.001**	***p* < 0.001**
MFR LAD	0.90	− 0.06 ± 0.26	0.95	− 0.08 ± 0.20	***p* = 0.010**	*p* = 0.196	***p* = 0.005**
MFR LCx	0.90	− 0.08 ± 0.29	0.97	− 0.08 ± 0.19	***p* = 0.001**	*p* = 0.903	***p* < 0.001**
MFR RCA	0.81	− 0.11 ± 0.33	0.96	− 0.08 ± 0.22	***p* < 0.001**	*p* = 0.276	***p* < 0.001**
MFR Total	0.92	− 0.05 ± 0.21	0.95	− 0.08 ± 0.19	*p* = 0.284	*p* = 0.217	*p* = 0.566

Significant values are in bold. MBF values are in ml/min/g.

In RET model, correlations of stress/rest MBF and MFR values obtained by two operators did not differ significantly between individual vascular territories. In 1CM model, however, correlations of stress/rest MBF and MFR values obtained by two operators in RCA vascular territory were significantly lower than analogous correlations in the entire myocardium (0.84 vs 0.91, *p* = 0.028 for stress MBF; 0.72 vs 0.84, *p* = 0.025 for rest MBF; 0.81 vs 0.92, *p* = 0.001 for MFR).

Some authors used standard deviation of the percentage difference between values in compared samples as an additional measure of precision of determined parameters—its values for MBF and MFR obtained by two different operators in both models are presented in Table 7, along with similar parameters reported in most recent literature.

**Table 7:** Standard deviation of percentage difference of MBF and MFR values obtained by two different operators in both models in this study compared with results reported in most recent literature^5,6,9^ for the whole myocardium (TOT).

	1 compartment			Net retention		
	Cichocki et. al.	Cuddy-Walsh et. al.	Wells et. al.	Cichocki et. al.	Cuddy-Walsh et. al.	Bailly et. al.
Program	4DM v2024	4DM v2017	FlowQuant	4DM v2024	4DM v2017	4DM*
Patients	107	31	30	107	31	70
TOT MBF Stress	12.5%	15.6%	16.4%	9.0%	11.3%	32.2%
TOT MBF Rest	12.1%	16.4%	12.7%	9.5%	12.4%	25.3%
TOT MFR	10.3%	20.2%	18.9%	9.4%	17.8%	18.9%

*Unknown version of 4DM, but considering the time of publication, Xeleris workstation at that time was most likely equipped with v2015 or v2017.

## Discussion

In Corridor 4DM v2024, the amount of necessary manual modifications in the first stage of dynamic SPECT MFR studies post-processing was significantly reduced. In our previous study using 4DM v2015, over 40% of studies (more often rest) required significant manual corrections of automatic alignment of images in terms of heart position and axis. At that time, we did not observe any clear relationship between the overall technical quality of the study and the occurrence of such problems. In 4DM v2024, however, the number of studies with such poor automatic positioning of the heart has dropped to just over 10% (with no significant differences between stress and rest studies) and currently such problems are observed practically only in the case of technically difficult studies—most often in patients with significantly decreased radiopharmaceutical uptake in the inferior wall (including permanent perfusion defects and stress-induced ischemia) combined with high activity of the radiopharmaceutical in the intestinal blood pool, as shown in Fig. 1b. In such situations, difficulties with automatic image alignment are probably unavoidable.

Improvement in automatic heart positioning significantly increased the correlation and decreased variation of MFR values obtained by two operators in RCA vascular territory, where we previously reported poor interobserver repeatability of these values^2^. Corrections to the software algorithm were made based on data of patients from group I, but similar processing repeatability was also achieved in a second, independent group of patients.

Despite such significant improvement in automatic alignment of heart images, correlations of MFR values obtained by different operators in group I in RCA territory using 1CM model, although clearly improved, were still significantly lower compared to such correlations in other vascular territories and the entire myocardium (0.85 vs 0.93/0.94, *p* = 0.0389/0.0137). Study by Cuddy-Walsh et. al. showed clear differences in the precision of values obtained using 1CM and RET models^6^. Therefore, in our work we also compared the performance of these two models, based on a much larger group of patients and using the newest 4DM v2024.

Mean values of stress/rest MBF obtained by the same operator in 1CM model were generally higher than in the RET model, while the opposite trend was observed for MFR values (although not all differences were significant—Table 5). For all parameters, the biggest differences were consistently observed in RCA territory, while no statistically significant differences were observed in LCx territory.

MBF and MFR values in 1CM model displayed worse inter-observer processing repeatability compared to RET model, except for the whole myocardium MFR (Table 6). In the 1CM model, the highest inter-observer variability and lowest correlations of all parameters were still consistently observed in the RCA territory, especially in the case of rest MBF. This was not the case in RET model, where variability and correlations were similar in all vascular territories and whole myocardium. In both models, however, slightly lower correlation coefficients of rest MBF compared to stress MBF were found. Conversely, stress MBF values displayed higher variability than rest MBF values.

Differences in correlations and variability of MBF and MFR values calculated in 1CM and RET models may, to a large extent, result from different methods of spillover correction used in both models. RET uses a constant correction for the entire myocardium, depending only on LV blood pool activity, while 1CM model uses more accurate correction of spillover effect (and partial-volume effect), calculated separately for each area of the myocardium. For high-resolution PET studies, more complex 1CM model may be a better option. However, in SPECT studies its precision may actually be worse^6^. Better repeatability of MBF and MFR values calculated using simpler RET model may be due to its lower susceptibility to variability resulting from manual heart motion correction. In this model, the only variable that directly affects the spillover correction is the activity in LV ROI. Differences in study processing between operators will still affect the activity measured in given myocardial segments, but the spillover correction will be much more stable than in 1CM model.

Improved automatic alignment of the heart images also improved precision (expressed as standard deviation of percentage difference of assessed values) of MBF and MFR values in both models, compared to similar parameter presented in recent literature (Table 7). However, this brief review of recent literature highlights the lack of standardization of dynamic SPECT studies assessment methodology. Bailly et. al. only noted the lack of patient motion correction but did not state if and how heart motion correction was carried out^5^. Wells et. al. were using different software^9^. Cuddy-Walsh et. al. were setting the valve plane at 50% of the activity in the septum in HLA projection, while in our studies we were setting it at 50% of the activity in the anterior wall in VLA view^6^. Due to the above-mentioned methodological differences, the comparison of this parameter between these studies has certain limitations.

In this study, we were relying on the same post-processing methodology we used consistently with 4DM v2015. However, we believe that 4DM v2024, with different interface and high resolution blood flow and MFR polar maps, gives room for some improvements to the methodology, that could increase the precision of the results. In our opinion, visual assessment of high-resolution blood flow polar maps could be an additional element of quality control. This could potentially help reduce the impact of spillover artifacts with better heart motion correction. While automatic LV ROI placement is the same in both versions of the 4DM software, position of the valve plane line, affecting the position of LV ROI, is also an aspect that needs some consideration. Setting this line at 50% of the activity in the anterior wall used in this paper (for consistency with our previous work) may be difficult in patients with prefusion defects in the basal part of LV anterior wall in one or both parts of the study. On the other hand, setting the valve plane at 50% of the activity in the septum may also lead to difficulties in case of perfusion defects in this area (commonly seen in patients with left bundle branch block for example). Comparison of different valve plane line setting methods needs further investigation, since it affects the final positioning of LV ROI, that greatly affects final MBF and MFR values.

Likewise, we consistently used the same radiopharmaceutical injection method as in our earlier work. Manual, rapid bolus injection method was chosen based on our experience with first-pass angiocardiography studies, where such injection method consistently results in good quality bolus TACs. Poor bolus quality, that led to exclusion of 7 patients from this study, was either consistently observed in stress and rest studies (suggesting a patient-related cause, eg. radiopharmaceutical flow being delayed by valves, rather than injection-related cause) or was caused by radiopharmaceutical leaking outside of the vein, possibly due to vein rupture in some cases. Using a slower, continuous injection method could potentially reduce the likelihood of rupturing veins, though it is unlikely to solve patient-related problems. Despite good quality of the majority of bolus TACs achieved using our method, use of automatic injector instead of manual injection in future studies may be worth a consideration, to assure the best possible repeatability of radiopharmaceutical administration.

While the precision and processing repeatability were improved, our study revealed significant differences between values calculated in 1CM and RET models (Table 5). It would be ideal to compare MBF and MFR values obtained in SPECT studies with the new, improved software with the gold standard—PET—to recalibrate both models and improve their accuracy. Lack of verification of MBF and MFR values with PET studies is the limitation of our study in this regard, but such assessment should be considered for future investigation.

Most of the patients included in our study were clinically verified by coronary angiography. Comparison of MBF and MFR values assessed after updating and optimizing post-processing methodology with the results of coronary angiography will be the subject of our further studies.

## Conclusions

1. In SPECT studies, the net retention model is characterized by better processing repeatability than 1 compartment model and, on average, generates lower MBF and higher MFR values.
2. Improved automatic positioning and orientation of heart images in Corridor 4DM v2024 significantly improved processing repeatability of MBF and MFR values based on dynamic SPECT data obtained on the CZT Discovery NM530c gamma camera.
3. Better standardization of dynamic SPECT MFR study processing methodology, in regard to LV valve plane setting and heart motion correction, is still necessary to achieve consistent values between different departments.
4. Recalibration of 1 compartment and net retention models with PET for use in SPECT studies should be considered.

### Supplementary Information

Supplementary Information.

## Data Availability

The datasets generated and analyzed during the current study are available from the corresponding author on reasonable request.
