# Supplementary material for: Higher processing repeatability of myocardial flow reserve calculated using net retention model compared to one compartment model in SPECT studies
Source: Sci Rep. 2024 Jul 19;14:16671. doi: 10.1038/s41598-024-67474-z (PMC11271556; doi:10.1038/s41598-024-67474-z)
Supplement: Supplementary file 1 — Supplementary Information. [file 41598_2024_67474_MOESM1_ESM.docx]

**Higher Processing Repeatability of Myocardial Flow Reserve Calculated Using Net Retention Model Compared to One Compartment Model in SPECT studies**

*Pawel Cichocki^1^, Anna Plachcinska^2^, Michal Blaszczyk^1^, Zbigniew Adamczewski^1^

^1^ Department of Nuclear Medicine, Medical University, Lodz, Poland

^2^ Department of Quality Control and Radiological Protection, Medical University, Lodz, Poland

Supplementary Materials

Formulas used in calculation of myocardial blood flow and myocardial flow reserve.

Net retention model (RET) is based on formula (1):

$R=\frac{\frac{1}{\left( t3-t2 \right)}\int_{t2}^{t3} Cm\left( t \right)-Sm*Ca\left( t \right)dt}{PV*CF\int_{0}^{t1} Ca\left( t \right)-Sb*Cm\left( t \right)dt}$ (1)

|  |
| --- |

where R – retention rate, PV – partial volume correction, CF – correction factor, Sm - spillover from blood pool to myocardium, Sb - spillover from myocardium to blood pool, t1 - upper time limit of blood pool integration, t2 and t3 - upper and lower time limit of tissue curve integration, respectively, Cm(t) – measured left ventricle myocardium radiotracer concentration, Ca(t) – left ventricle blood pool radiotracer concentration. Following preset values were used in this study: PV = 0.67, CF = 1, Sm = 0.2, Sb = 0, t1, t2 = 60s and t3 = 120s, in accordance with the default settings of our 4DM v2024 software.

Sb for ^99m^Tc-MIBI and ^99m^Tc-tetrofosmin, most commonly used in SPECT, is assumed to be negligible and is typically set to 0. PV and Sm parameters are also constant, preset in the system, so they are the same for every patient, which may lower the accuracy of this model. Also note that spillover correction will be the same for each segment of the myocardium, since Sm is only modified by blood pool TAC, Ca(t), and does not depend on Cm(t).

One tissue compartment model (1CM) uses more complex calculations, relying on a non-linear fit of K_1_, DV, MBV and MVV parameters to dynamic image data, using formulas (2) and (3):

| $C_{T}\left( t \right)=K_{1}e^{-\left( \frac{K_{1}}{DV} \right)t}⮾ Ca\left( t \right)$ (2)  $Cm\left( t \right)=\frac{1}{\left( t2-t1 \right)}\left[ \left( 1-TBV \right)P_{t}\int_{t1}^{t2} C_{T}\left( t \right)dt+TBV\int_{t1}^{t2} Ca\left( t \right)dt \right]$ (3) |
| --- |

where C_T_(t) – total myocardial tissue radiopharmaceutical concentration, K_1_ –uptake rate constant, DV – distribution volume, TBV – total fractional blood volume (radiopharmaceutical concentration in blood compartment within left ventricle contour, which accounts for both partial volume and spillover effects), P_t_ – tissue density, t1 and t2 – start and end of data acquisition, Cm(t) – measured left ventricle myocardium radiotracer concentration, Ca(t) – left ventricle blood pool radiotracer concentration. ⮾ symbol represents convolution of two functions.

In this model, fractional blood volumes - MBV and MVV parameters is accounting for both partial volume and spillover (both into and out of myocardium) effects. This means these effects are calculated for each patient independently, increasing the overall accuracy of the model. They are also calculated for each segment of the myocardium separately, since unlike Sm in RET model, MBV and MVV are modified by radiotracer concentration in both blood pool - Ca(t) and myocardial tissue – C_T_(t).

R and K_1_ parameters are related to MBF in a similar way, through Renkin-Crone extraction fraction - formula (4). A and B parameters are predetermined empirically and preset in the software for each model.

| $K_{1}=MBF*\left( 1-A*e^{-\left( \frac{B}{MBF} \right)} \right)$ (4) |
| --- |
